# On the Long-term Stability of Clines in Some Metabolic Genes in *Drosophila melanogaster*

**DOI:** 10.1038/srep42766

**Published:** 2017-02-21

**Authors:** Rodrigo Cogni, Kate Kuczynski, Spencer Koury, Erik Lavington, Emily L. Behrman, Katherine R. O’Brien, Paul S. Schmidt, Walter F. Eanes

**Affiliations:** 1 Department of Ecology and Evolution, Stony Brook University, Stony Brook, New York, 11794 USA; 2 Department of Biology, University of Pennsylvania, Philadelphia, PA, USA

## Abstract

Very little information exists for long-term changes in genetic variation in natural populations. Here we take the unique opportunity to compare a set of data for SNPs in 15 metabolic genes from eastern US collections of *Drosophila melanogaster* that span a large latitudinal range and represent two collections separated by 12 to 13 years. We also expand this to a 22-year interval for the *Adh* gene and approximately 30 years for the *G6pd* and *Pgd* genes. During these intervals, five genes showed a statistically significant change in average SNP allele frequency corrected for latitude. While much remains unchanged, we see five genes where latitudinal clines have been lost or gained and two where the slope significantly changes. The long-term frequency shift towards a southern favored *Adh S* allele reported in Australia populations is not observed in the eastern US over a period of 21 years. There is no general pattern of southern-favored or northern-favored alleles increasing in frequency across the genes. This observation points to the fluid nature of some allelic variation over this time period and the action of selective responses or migration that may be more regional than uniformly imposed across the cline.

The study of geographic variation is an important approach in the inference of adaptation in evolutionary genetics, and work in *Drosophila melanogaster* has played a central role in these investigations over many decades. This special attention is motivated by the complex biogeographic history of this species that involves the interrelationships between migration and local adaptation during a global colonization process, across different time scales. In that effort, many geographic clines in *Drosophila melanogaster* have been reported and these encompass a wide range of phenotypic and genetic variation that includes physiological, life-history, and morphological traits, chromosomal inversions, and molecular polymorphisms^1^,^2^,^3^,^4^. A vast majority of this work has investigated populations on the east coast of Australia^5^ and the eastern US^6^,^7^,^8^.

With respect to the study of molecular polymorphism, the discovery of allozyme clines was first systematically explored by John Oakeshott and colleagues^9^,^10^,^11^,^12^,^13^ in a series of papers on Australian, North American, and European collections in the early 1980s. These studies found a number of parallel independent examples of clines in allozyme polymorphisms in the northern and southern hemispheres. These early studies supported the theme of an underlying commonality of clinal selection regimes in both the Australian and North American populations, and these have formed the working hypothesis and context of many studies that suggest these latitudinal clines represent evidence of natural selection acting after colonization and population spread^7^,^14^,^15^,^16^,^17^.

Out of these studies emerges the question as to how stable is the observed geographic variation over both short and long time scales? On the short-time scale, there is evidence that some variation can change seasonally^18^,^19^,^20^,^21^. On the long-time scale, understanding the stability of clines and geographic variation is particularly relevant because the potential selection that maintains polymorphism can be globally or locally changing, and processes such as admixture can always confound interpretation. There is an emerging observation that these *D. melanogaster* populations and the associated clines in North America and Australia are the result of admixtures of European temperate populations and their ancestral tropical African populations through independent invasive colonizations^22^,^23^,^24^,^25^. Therefore, a major challenge is to identify which clinal polymorphisms are maintained by ongoing selection and which are the result of admixture and possibly transient neutral variation.

One approach that can help to answer this question is to compare the stability of clines over long time periods, such as over a decade. There is little data on the stability of clines or the local stability of allele frequencies in *D. melanogaster*. Using the reported data from Oakeshott collections in Australia around 1980, Umina and colleagues^26^ examined the state of several clines reported from collections from 2000–2002 across the same latitudinal range, spanning a period of about two and half decades. They included clines for *Adh* and *Gpdh* and the inversion polymorphisms for *In(2L)t* and *In(3R)Payne*. They found notable change for *Adh* and *In(3R)P* with the tropically-favored allele increasing over time. Cogni and colleague^19^ looked at the clinal SNP polymorphisms for the diapause-associated *cpo* gene^27^ and found these clines were relatively unchanged compared to the latitudinal clines reported 13 years earlier. In this report, we have the unique opportunity to revisit a number of clines of molecular polymorphisms in some metabolic genes reported in previous studies of *Drosophila melanogaster* along the east coast of the US. These data are from collections made in 1997 and reported in Sezgin *et al*.^8^ and collections from the 2009–2010 used in the analyses by Lavington *et al*.^28^. Our main goal is to describe patterns of changes over a 13-years interval in clines in 15 metabolic genes.

## Results

### Patterns of clinal change over a 13-years period vary among genes

We compared clines in collections along a broad latitudinal gradient in the Eastern US made in 1997 and 2009/2010. We have ten collections made in 1997 that were reported in Sezgin *et al*.^8^ and Verrelli and Eanes^29^, and 18 collections made in 2009/2010 that were reported in Lavington *et al*.^28^(Supplemental Table 1). To provide further context we also include SNP frequency estimates for three European populations that extend farther into temperate latitudes (see methods). These studies addressed cline variation in genes involved in central metabolism. We looked for clinal patterns of 21 SNPs distributed in 15 metabolic genes for which we have data for both time points (1997 and 2009–2010). We used a linear model (see methods) that tested the effect of sampling period and latitude on SNP frequency. Specifically, we tested for the presence of a cline (significant regression with latitude) in each sampling period and on all collections combined (Table 1). We also tested for a difference in allele frequency between the two sampling periods and for a difference in the slope of the regression with latitude between the two collection periods (Table 1). Power analyses (see methods) showed that we have good power to detect overall SNP frequency changes between samples, reduced power to detect changes in slope between years, and for the detection of significant clines for each individual SNP the 1997 samples have about half the power of the 2009/2010 samples (Supplemental Fig. 1). The fact that we found many significant clines in the 1997 samples, even with reduced power, indicates that clines in metabolic genes are pervasive. Genes that lie inside the cosmopolitan inversions are indicated in Table 1. Of the 21 SNPs, six were significantly clinal in both sampling time points and eight were not clinal in either year. For seven SNPs a significant cline was observed in only one year, and in five of these cases the clines observed in 1997 were lost in 2009/2010, despite the higher power to detect clines in the 2009/2010 collections (Supplemental Fig. 1). Allele frequencies did not show a statistically significant change for 15 out of 21 SNPs. For six SNPs (in five genes) there was a significant change in average SNP allele frequency corrected for latitude, in three of these five genes the southern associated allele increased in frequency. The overall results for all 15 genes are listed in Table 1. We discuss significant cases below.

### Southern *trehalase (Treh)* allele increased in frequency in northern populations

**Figure 1:**
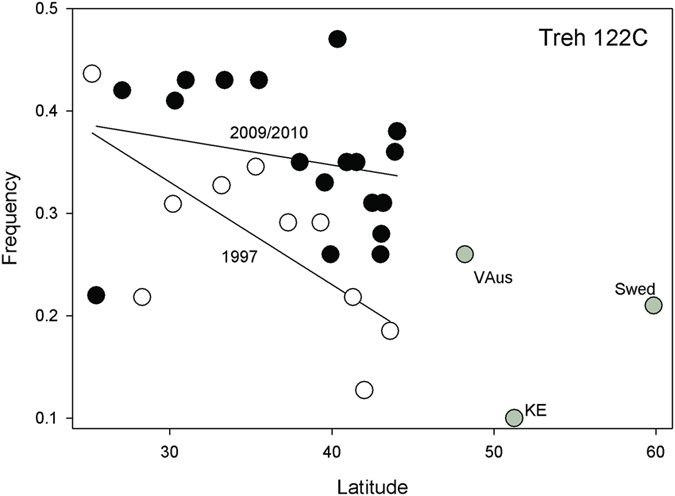
Plot of *Treh*^*122C*^ SNP frequencies in 1997 (open circles) and 2009/2010 collections (black circles) against latitude. The European collections are provided (grey circles).

In the 1997 collections, Sezgin *et al*.^8^ reported a cline in the *Treh*^*122C*^. which is an amino acid (V41A) polymorphism. Twelve years later we observe a clear but non-significant shift (*t*_*26*_ = 1.352, *P* < 0.0941) in the cline that reflects an overall 10% increase in latitude-adjusted frequency of the southern associated *Treh*^*122C*^ *allele (t*_*26*_* = 3.45, P* < 0.0021). This shift appears driven by an increase of about 15% of the southern allele in northern populations over this period (Fig. 1). This gene lies near the distal breakpoint of inversion *In(2R)NS*, but this inversion is extremely rare in northern latitudes.

### Significant clines in *phosphoglucomutase (Pgm)* were lost

**Figure 2:**
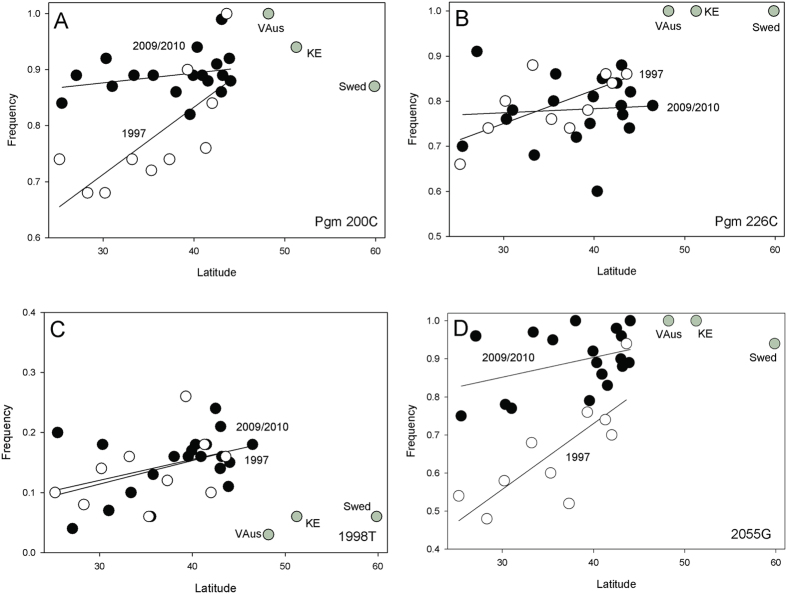
Plot of *Pgm* SNP frequencies in 1997 (open circles) and 2009/2010 collections (black circles) against latitude. (**A**) *Pgm*^*200C*^, (**B**) *Pgm*^*226C*^, (**C**) *Pgm*^*1998T*^, (**D**) *Pgm*^*2055G*^. The European collections are provided (grey circles).

In the 1997 collections, we reported latitudinal clines in a number of SNPs^29^. We reexamine four here: one synonymous and three replacement SNPs. In 1997, *Pgm*^*200C*^ (syn), *Pgm*^*226C*^(V52A), and *Pgm*^*2055G*^(V484L) had significant clines. *Pgm*^*1998T*^ (T465S) did not. In 2009/2010, there are significant changes in these SNPs. Although there are larger sample sizes, none show significant clines (Fig. 2A–D) and *Pgm*^*200C*^ and *Pgm*^*2055C*^ each show an overall but non-significant (*t*_*26*_ = 1.683, *P* < 0.0522; *t*_*26*_ = .848, *P* < 0.202) increase in the latitude-adjusted frequencies of northern-associated alleles into the southern populations. Both alleles show overall significant increases in frequency (*t*_*26*_ = 2.37, *P* < 0.0261; *t*_*26*_ = 4.88, *P* < 0.0001). The *Pgm*^*200C*^ *and* Pgm^2055G^ alleles are in linkage disequilibrium with each other^29^, so their shift is corroborated here. *Pgm* is inside the inversion *In(3L)P*, however shifts in the inversion frequency cannot explain the increase in frequency of *Pgm*^*200C*^ *and* Pgm^2055G^ since they have the opposite association inside the inversion.

### Significant cline in *phosphoglucose isomerase (Pgi)* was gained

**Figure 3:**
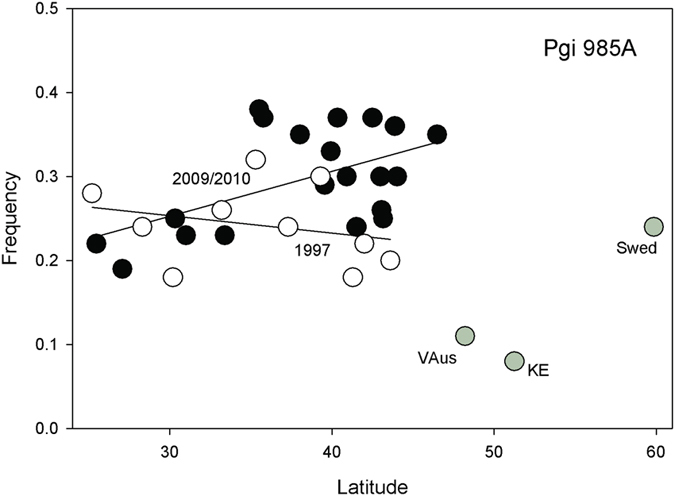
Plot of *Pgi*^*985A*^ SNP frequencies in 1997 (open circles) and 2009/2010 collections (black circles) against latitude. The European collections are provided (grey circles).

The amino acid polymorphism in *Pgi*^*985A*^ (V329I) possessed no significant cline in 1997, but there is now a significant cline in the 2009/2010 sample (Fig. 3; *t* = 1.715, *p* < 0.0491). *Pgi*^*985A*^ is defined as a southern favored allele since its frequency is lower in Europe than in the North American populations. Notably, its frequency has increased by 4% overall (*t* = 2.32, *p* < 0.0291) and by about 10% in northern populations. It is well outside the *In(2R)NS* inversion, which is extremely rare in northern latitudes.

### Significant cline in glyceraldehyde-3-phosphate dehydrogenase (Gapdh1) was lost

**Figure 4:**
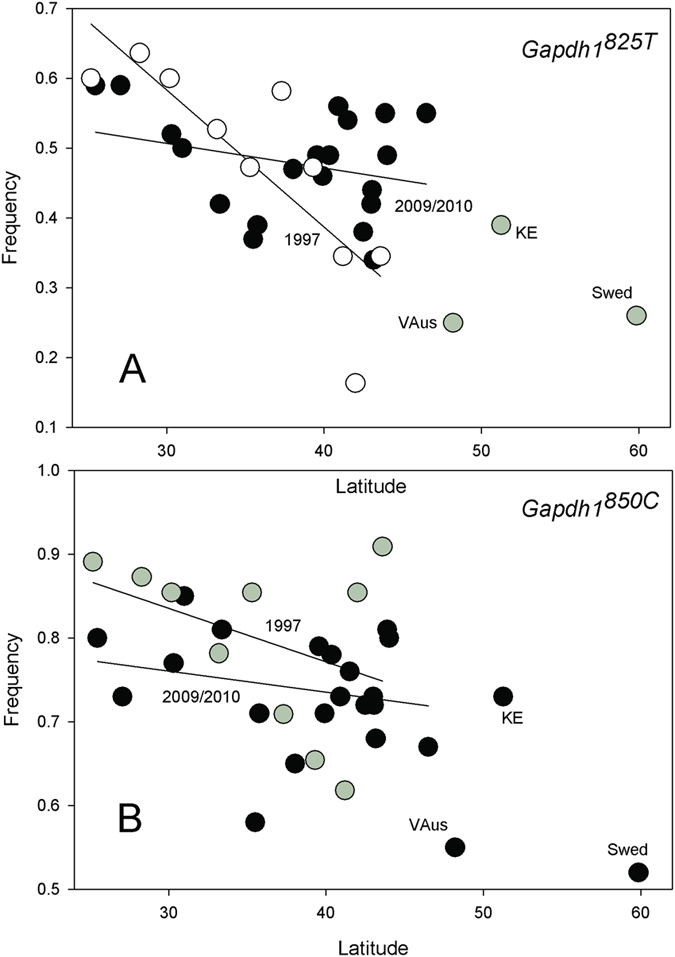
Plot of *Gapdh1* SNP frequencies in 1997 (open circles) and 2009/2010 collections (black circles) against latitude.

Two SNPs in *Gapdh1* were screened in 1997 and there was a significant cline in the *Gapdh1*^*825C*^ allele. There is also a significant shift in the clines between time intervals (*t* = 1.924, *p* < 0.0327). We fail to see this cline in 2009/2010 (Fig. 4). *Gapdh1* is well outside the *In(2R)NS* inversion, which is extremely rare in northern latitudes. This was interpreted as a slight, albeit significant, shift towards the more southern based allele.

### Northern *phosphoglucokinase (Pgk)* allele increased in frequency

**Figure 5:**
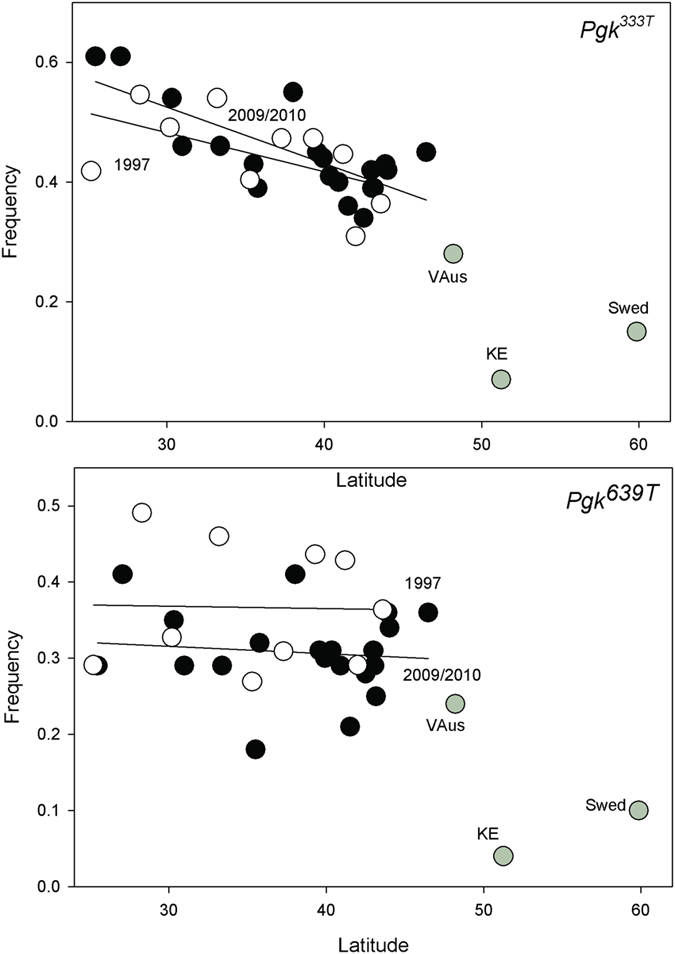
Plot of *Pgk*^*333T*^(**A**) and *Pgk*^*639T*^ (**B**) SNP frequencies in 1979/1980 (open circles) and 2009/2010 collections (black circles) against latitude. The European collections are provided (grey circles).

Two SNPs in *Pgk* were screened in 1997 and there was a significant shift in the *Pgk*^*639T*^ allele (Fig. 5). This was interpreted as a slight, albeit significant, shift towards the more northern based allele.

### Southern *glucose-6-phosphate dehydrogenase (G6pd)* allele increased in frequency

We are able to examine allozyme clines for *G6pd-F* reported in Oakeshott *et al*.^12^. This is SNP 1145 in Lavington *et al*.^28^ and the amino acid polymorphism P382L^30^. Oakeshott *et al*.^12^ also includes a set of data for three localities collected in the 1970s by Berger^31^. The data are sampled largely in 1979/1983 and permit a look at potential changes over 29–31 years. In 1983, there was a significant partial regression, but the linear regression in our analysis in 2009/2010 is not significant. In 1979/1983, the frequency of the *F* allele was nearly 100% in Europe and fixed in many samples, although our three collections for Sweden, England, and Austria show a very low frequency from recent collections. *G6pd* is X-linked and not associated with any inversion.

**Figure 6:**
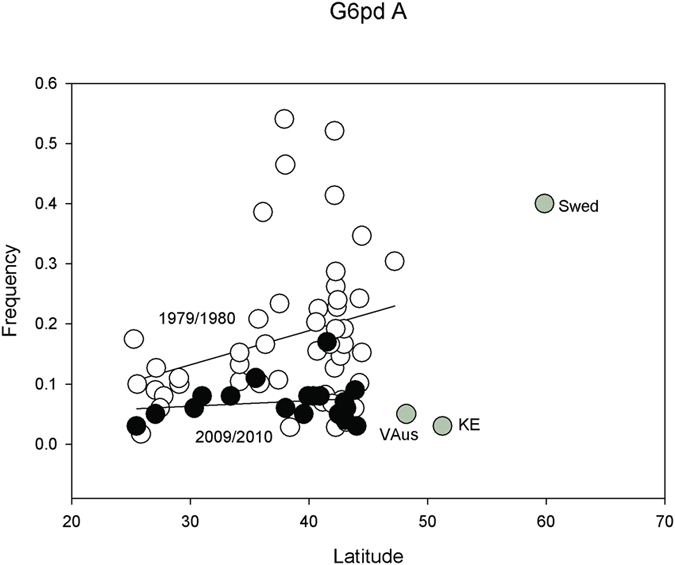
Plot of *G6pd*^*F*^ SNP frequencies in 1979/1980 (open circles) and 2009/2010 collections (black circles) against latitude. The European collections are provided (grey circles).

There is a dramatic drop in the overall frequency in the *Gpdh*^*F*^allele over this three-decade interval (*t* = 4.23, *p* < 0.0001; Fig. 6). Earlier collections showed many northern samples where the *F* allele is found in the ranges of 10 to 50%; these have disappeared in 2009/2010. All change is in the direction of increasing frequency of the southern associated allele.

### Southern *phosphogluconate dehydrogenase (Pgd)* allele increased in frequency

**Figure 7:**
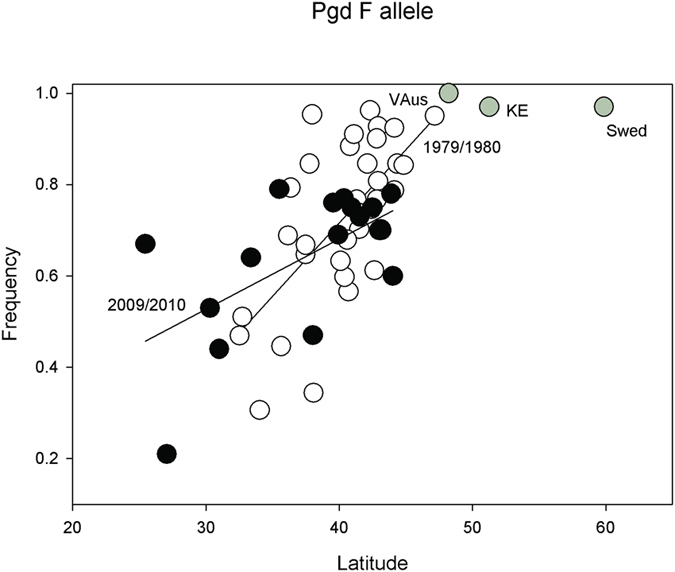
Plot of *Pgd*^*F*^ SNP frequencies in 1979/1980 (open circles) and 2009/2010 collections (black circles) against latitude. The European collections are provided (grey circles).

The cline in the *Pgd*^*F*^ allele (Gln/Lys polymorphism) reported by Oakeshott^12^ is present decades later (Fig. 7). Both temporal samples show highly significant latitudinal clines (Table 1). There is a clear paucity of samples with *Pgd*^*F*^ >0.80 in recent collections (perhaps consistent with the influx of southern favored alleles). There is an increase in the frequency of the southern favored allele in northern latitudes.

### No changes in *alcohol dehydrogenase (Adh)* cline in North America over 21 to 27 years

**Figure 8:**
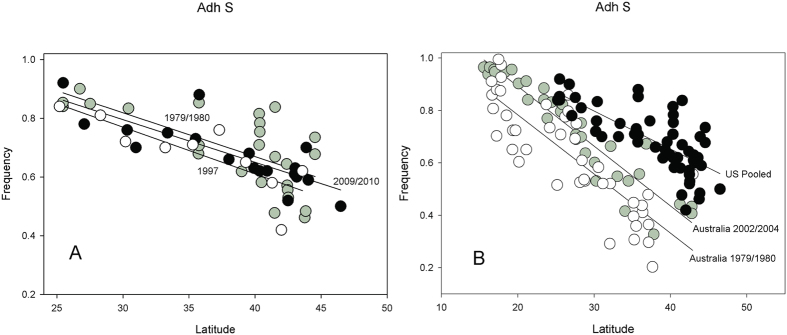
Plot of *Adh*^*S*^ SNP frequencies against latitude. (**A**) Eastern US collections in 1979/1980 (open circles), 1997 (grey circles) and 2009/2010 (black circles). (**B**) Plot of Australian samples from Umina (Umina *et al*.^26^) samples in 1979/1980 (open circles) and 2002/2004 (grey circles) against the pooled collection for eastern US all years (black circles).

One of the best studied clines in *D. melanogaster* is the ADH allozyme polymorphism that represents a Thr/Lys amino acid change. We can reexamine the *Adh*^*S*^ cline in the eastern US using data collected from 1983 to 1988 by Berry and Kreitman^32^. Using our *Adh* data (2009/2010) this spans an interval of 21 to 27 years. There is no significant *Adh*^*S*^ frequency difference over this interval (*t* = 1.17, *p < *0.2476) and there is no significant difference in the slopes (Fig. 8A). While the difference in mean change in *Adh*^*S*^ allele in North America is not significantly different from zero, nonetheless, it is significantly different from a 10% change observed in the Australian samples. Our power analyses indicates that we have 100% power to detect a 10% change in frequency between 1993 and 2009/2010, and even the detection of a 5% change in frequency has a 93% power (Supplemental information). We can also place our US clines in the context of the Australian clines^26^ (Fig. 8B). We see the slopes are significantly different (*t* = 1.761, *p* < 0.045), but more importantly there is a highly significant displacement in latitude-adjusted mean *Adh*^*S*^ frequency (*t* = 8.97, *p* < 0.0001).

## Discussion

In this report, we first examined 15 genes from 10 US samples that were collected in 1997 by Brian Verrelli and the data reported by Sezgin and colleagues^8^, and compared these results to a new collection of 18 samples made in 2009–2010 across the same latitudinal range^28^. In the latter collection, we also sampled the same amino acid polymorphisms in *G6pd* and *Pgd* that were examined in North American collections made in the early 1980s^12^. Finally, we were able to screen and contrast the *Adh*^*S*^ allele frequencies in the 2009/2010 to collections made not only in 1997, but also the late 1980s by Berry and Kreitman^32^ and compare these clines to those reported for the latitudinal ranges studied in Australia. If we assume 10 generations per year in the southern US and six generations in the northern US, our 1997 to 2009/2010 interval spans 75 to 120 generations of potential change.

When we combine the overall data from both US collections, there are clines observed in 11 of 15 genes. This is consistent with the large number of clines reported in *D. melanogaster* in North America and Australia. Of the 20 SNPs in 15 genes, we see seven cases where a cline was reported in only one sample, but not both samples. In two cases (*Pgi, Gapdh1*) there is a statistically significant shift in cline slope. While most of the genes show little significant change over the 12 to 13-year time interval here, this implies that even over these relatively short time scales some geographic variation can be fluid.

The different SNPs are likely responding to different processes. While some clines are likely kept by spatial differences in selection^19^,^20^,^33^, some neutral polymorphism may be clinal by the result of admixture of African and European populations^24^,^25^. Some clines may first appear as the result of admixture, but spatial differences in selection may maintain them, such as in the case of European adapted alleles being selected against on the populations closer to the tropics. The problems associated with isolation-by-distance in the generation of spatial patterning has been well emphasized^34^,^35^. Long term data on the stability of clines as reported here can in principle help to discern among these different hypotheses. For example, if clines are the result of admixture, gene flow will reduce the differences among populations over time and the clines will be lost. Changes in clines for neutral SNPs should be relatively slow over this number of generations, but other measures of admixture, such as linkage disequilibrium, may be predicted to change much more rapidly over these time scales. Although the time of colonization of *D. melanogaster* is believed to be about 150 years in North America^36^, the colonization of the tropical New World may be older and the assumption of single point colonization is unknown; furthermore, the precise origins of admixture populations are also not known.

The steep *Pgm* clines reported in 1997 for SNPs *Pgm*^*129C*^ and *Pgm*^*2005C*^ are absent in 2009/2010. In both cases, there is a significant increase in the temperate-favored northern allele in southern parts of the cline. Both SNP alleles are in linkage disequilibrium so we expect correlated changes. The reported clines in the *Treh* amino acid polymorphism and *Gapdh1* seen in 1997 appear absent in the 2009/2010 collection and a cline for *Pgi*, not seen in 1997, is now observed. Both of these latter cases involve increases in the southern-based allele in northern latitudes. We should also emphasize that both *Gapdh1* and *Pgi* are on the 2R arm which carries the *In(2R)NS* inversion. This inversion is very rare in all eastern US populations^6^,^8^,^37^, including our 1997 populations and is an unlikely cause of change in these genes. The genes that lie inside the cosmopolitan inversions are listed in Table 1.

Both the *G6pd*^*F*^ and *Pgd*^*F*^ alleles were examined in collections from the early 1980s^12^. These collections permit temporal comparisons of about three decades or perhaps 200 to 300 generations. *G6pd* shows a notable change in overall frequency with the *F* allele polymorphism appearing to disappear from northern populations. While there is no significant cline, the global pattern clearly shows the *F* allele to be temperate-favored (it was nearly fixed in European populations in 1979/1980), while the ancestral *S* allele is the most common in tropical regions. This is a possible case of invasion of the tropically-favored allele over this three-decade span, or simply the selective removal of the temperate allele. This appears to have also happened in Europe where earlier estimates found it effectively fixed^12^.

The *Pgd*^*F*^ allele shows the same cline after 30 years, with a slight, but not significant, shift in overall frequency towards the southern-favored allele. There is again a very notable drop in the frequency of the temperate favored *F* allele above 35 degrees N latitude. There were many populations with frequencies >80% in the earlier interval, but in 2009/2010 there were none. The European populations are nearly fixed for the *F* allele^12^ and this allele has been shown in a genome scan of Netherland samples of the X chromosome to be associated with an apparent selective sweep in this region. This sweep is centered on the *Pgd* locus^38^, and may be the cause because of direct selection or be hitchhiking on other genes in the small region of 60.5 kb, and estimated to hold seven genes.

Several interesting features emerge from the comparison of the *Adh*^*S*^ allele clines. First, the US samples do not show the same latitude-controlled *Adh*^*S*^ allele frequency shift reported by Umina and colleagues^26^. The *S* allele frequency is not significantly different among the three-time collection points on the east coast of the US. The data of Berry and Kreitman^32^, collected 21–22 years earlier than our 2009/2010 collection, is comparable to the Australian study that spanned 23–25 years. The eastern US latitudinal range is smaller, spanning 20 degrees of latitude compared to nearly 30 degrees for the Australian collection, and extends farther into temperate latitudes, but it is doubtful that this causes the lack of a shift in US populations. Second, what is particularly interesting is the displacement of the Australian and eastern US latitudinal clines by 30% at the higher latitudes. This might suggest that temperatures across the US range are higher; however, mean annual temperatures in the higher latitudes are effectively identical between Australian and the eastern US localities at the same latitude. This disparity in Australian and US latitudinal clines should dispel the role of temperature as a sole environmental parameter driving selection of the *Adh* amino acid polymorphism via simple temperature dependent catalytic function. Umina *et al*.^26^ could not identify a single climatic variable that could explain the shift in the cline, suggesting that a combination of climatic factors, possibly including temperature was responsible. Given the large number of studies finding evidence for phenotypic differences for this polymorphism, it is unlikely that natural selection is not a factor in maintaining this polymorphism in *Adh*. However, as emphasized by Bradshaw and Holzapfel^39^, latitude is not just reflecting average temperature, but is a proxy for numerous other environmental parameters that likely differ across these continents at these latitudes.

There is interest in studies of long-term changes in using the genetic composition of *Drosophila* populations, particularly as they serve as proxies for climate-driven change. There are cases where collection data exists from earlier decades^40^,^41^,^42^. Most of this work has focused on chromosomal inversions for which there exists not only data on older collections^40^, but also known associations with colonization events, such as *Drosophila subobscura* in the New World^43^. There is also a growing appreciation of the extent of even short-term seasonally driven shifts in inversion frequencies^44^,^45^, something^46^ recorded in *Drosophila pseudoobscura* some decades earlier. In *D. melanogaster* the In(3R)Payne cline in Australian east coast has shifted in position (latitudinal intercept) in 20 years, presumably due to climate changes^26^,^47^, but in North America the same inversion has remained invariant across ∼40 years, without any changes in clinal slope or intercept^48^. Interestingly, the cline in In(2L)t has shifted upwards in latitude, and the cline in In(3L)P has shifted downwards and become more shallow in North America, while clines on both these inversions have remained temporally stable in Australia^48^. The data here are individually of interest, but still insufficient to reach any general conclusions about increases in northern or southern alleles as a response to climate change or shifts in admixture over this time interval. Future studies looking at genome wide patterns of clinal variation at different time intervals are needed.

The implications of this study of a sample of central metabolic genes suggest that clines and geographic variation can be fluid over decadal scales. Some of these differences may simply reflect variation consistent with repeated geographic samples, and others appear as statistically significant shifts in allele frequency. This is also strongly suggested from seasonal data that implies strong local selection on some genes^18^,^19^,^20^. In both latitudinal samples the tendency is for midseason samples to be collected in north and late season collections made in the south, although there was no significant effect of collection date on the clines reported in Lavington *et al*.^28^. We do not know if the observed shifts reflect long-term directional changes or rather recurrent fluctuations in allele frequencies under natural selection or shifts in the genome proportions of European and African ancestry. Given the local structure where populations are likely reseeded from local refugia and subsequently subjected to high gene flow, one might expect regional shifts rather than systematic responses at large across the entire cline. There are long-term average global changes consistent with responses to climatic change, but much year to year change is local or regional. The data here are far too limited to expand questions to larger contexts, such as the functionally connected pathway responses as initiated in Lavington *et al*.^28^. The clear question is why are some gene frequencies relatively stable while others are shifting? For example, *Treh, G6pd, Pgi*, and *Pgd* are all closely localized in the top of the metabolic pathway and all appear to show an increase in southern-favored allele, but *Pgm* which is also at the top of the pathway shows the opposite pattern. This type of question directed at physiological context must await a more highly resolved study of many more genes. Nonetheless, the study of shifts in genetic variation in short and long-term contexts opens a new dimension to the study of natural selection and population structure.

## Methods

### Populations sampled

The clines for the 10 latitudinal collections from 1997 were reported by Sezgin *et al*.^8^ and Verrelli and Eanes^29^. The 2009/2010 cline data are described by Lavington *et al*.^28^. These data represent 18 collections made across the same latitudinal range as the 1997 collections. Many collections come from identical localities (Supplemental Table 1). The data points for Raleigh (collected 2005) and Sudbury, ON (collected in 2008) have been dropped from the analysis. The data used by Sezgin *et al*.^8^ for the *Adh, Pgd*, and *G6pd* genes comes from earlier work^12^,^32^, and these genes were resampled in 2009/2010 collections. We also include SNP frequency estimates for three recent collected European populations (Uppsala, Sweden, latitude 59.86; Kent, England, latitude 51.19; and Vienna, Austria, latitude 48.20) to provide further context. These are not included in any statistical analysis, but in most cases the European populations fall along the latitudinal regression trends.

### Statistical analyses

The first statistical analysis uses a model, Yi = μ + αj + βXk + ε, where *Y*_*i*_is the *i*th dependent allele frequency, *α* is the effect of the sampling interval *j*, and *X*_*k*_ the *k*th latitude to test for interval effects. The changes in overall frequency difference between sample intervals and slopes of the regression against latitude were tested by *t*-test. Analysis was carried out in JMP^®^ 11.2 on arcsine transformed frequencies^49^. The power of these tests was evaluated by power analyses (Supplemental Fig. 1). Our assessment of a cline depends on the significant presence of a linear regression of arcsine transformed frequency within year, or the combined years (in which each collection was treated as an independent point). This regression and its slope determine the tropical- or southern-biased allele in each SNP pair. The presence of a cline in any SNP places that locus in the clinal class.

### Allele frequency estimation

The allele frequency estimates come from several methodologies including allozymes, restriction site polymorphism counts, base-specific Sanger sequencing for the 1997 samples, and bulk pyrosequencing peak estimation for the 2009/2010 samples. The bulk pyrosequencing peak estimation results in very accurate estimates of allele frequencies. We examined the correlation between the “true” input DGRP line SNP frequency estimates (based on original whole-genome sequences of individual inbreed lines^50^) with the estimated value in the bulk DNA preparation from that DGRP collection. The correlation was *r* = 0.98, and there is clearly no systemic bias in the estimation (Supplemental Fig. 2).

## Additional Information

**How to cite this article:** Cogni, R. *et al*. On the Long-term Stability of Clines in Some Metabolic Genes in *Drosophila melanogaster.* *Sci. Rep.* **7**, 42766; doi: 10.1038/srep42766 (2017).

**Publisher's note:** Springer Nature remains neutral with regard to jurisdictional claims in published maps and institutional affiliations.

## Supplementary Material

Supplemental Information

Supplementary Dataset 1

Supplementary Dataset 2

Supplementary Dataset 3

Supplementary Dataset 4

Supplementary Dataset 5

Supplementary Dataset 6

Supplementary Dataset 7

Supplementary Dataset 8

Supplementary Dataset 9

Supplementary Dataset 10

Supplementary Dataset 11

Supplementary Dataset 12

Supplementary Dataset 13

Supplementary Dataset 14

Supplementary Dataset 15

## Figures and Tables

**Table 1:** Summary of individual *t*-tests of the significance of collection year (*t*_yr_) on latitude controlled mean frequency, clines with latitude with pooled years (*t*_lat_), separate year tests of slopes (*t*_1997_ and *t*_2009/2010_) and test of the difference in slopes between years (*t*_Δslopes_).

Gene	SNP	*t*_yr_	*t*_lat_	*t*_1997_	*t*_2009/2010_	*t*_Δslopes_	*P*
*Pgm*^3^	200C	2.43*	2.59*	3.58***	0.82	1.68	0.052
	226C	0.78	1.41	1.96	0.32	0.98	0.168
	1998T	0.13	2.18*	1.34	1.83	0.02	0.491
	2055G	4.88****	2.96**	2.6***	1.47	0.85	0.201
*Treh*^2^	122 C ()	3.45***	2.72*	2.87**	0.76	1.35	0.094
*Gdh*^4^	986A(Y/F)	0.70	5.4****	4.62****	2.84**	1.40	0.086
*Pgi*	985A(V/I)	2.32*	1.07	0.83	2.88**	**1.72**	**0.049**
*Gapdh1*	825C	1.31	4.76****	2.43*	1.26	**1.92**	**0.032**
	850C	1.59	1.54	1.47	0.62	0.57	0.286
*Pgk*^1^	333T	1.09	4.89****	2.32*	5.04****	0.85	0.201
	639T	2.18*	0.45	0.05	0.055	0.21	0.410
*UGPase*^3^	49T	0.7	5.11****	3.44***	3.98****	0.34	0.374
*Men*	881C	2.03	1.77	1.22	1.31	0.07	0.473
*Pglym78*	255T	0.97	1.67	0.86	1.62	0.21	0.419
*G6pdh*	1145(A)	4.23****	1.08	1.24	0.09	0.51	0.307
*TreS*^1^	2409G	0.33	2.09*	0.67	2.6*	0.74	0.232
*Eno*^1^	222A	0.77	2.54*	1.93	1.66	0.39	0.351
	30G	0.31	4.52****	3.38***	3.02**	0.62	0.227
*Pgd*	F	0.86	5.91****	4.95****	2.66*	1.51	0.071
*Adh*^1^	F	1.17	6.75****	5.64****	4.12****	0.31	0.338
*Gpdh*^1^	S	0.95	3.05**	2.777****	1.47	0.94	0.177

1*In(2L)t;*^2^*(In2R)NS;*^3^*In(3 L)P;*^4^*In(3 R)P.* Inside or associated with inversion.

*p < 0.05; **p < 0.01; ***p < 0.005; ****p < 0.001.
