# Supplementary material for: On the Long-term Stability of Clines in Some Metabolic Genes in Drosophila melanogaster
Source: Sci Rep. 2017 Feb 21;7:42766. doi: 10.1038/srep42766 (PMC5318857; doi:10.1038/srep42766)
Supplement: Supplemental Information [file srep42766-s1.pdf]

SUPPLEMENTAL INFORMATION

ON THE LONG-TERM STABILITY OF CLINES IN SOME METABOLIC GENES IN *DROSOPHILA MELANOGASTER*.

Rodrigo Cogni *[1,3], Kate Kuczynski[1], Spencer Koury[1], Erik Lavington[1], Emily L. Behrman[2], Katherine R. O'Brien[2], Paul S. Schmidt[2], and Walter F. Eanes[1]

[1] *Department of Ecology and Evolution, Stony Brook University, Stony Brook, New York 11794.* [2] *Department of Biology, University of Pennsylvania, Philadelphia, PA.*

[3]*Current Address; Departamento de Ecologia, Universidade de São Paulo, Brazil*

**Corresponding Author: R Cogni,* rcogni@usp.br

Supplemental Table 1. Samples used in the study.

Locality	Latitude	1997 Month of Collection	1997 # chromosomes[1]	2009/2010 Month of Collection	2009/2010 # chromosomes[1]
Bowdoin, ME	44.03	-	-	Oct 2009	172
Shoreham, VT	43.89	-	-	Sep 2009	200
Whiting, VT	43.86	Aug 1997	55	-	-
Marion, NY	43.16	-	-	Sep 2009	184
Newark, NY	43.05	-	-	Sep 2009	150
Putney, VT	43.00	-	-	Oct 2009	200
Harvard, MA	42.50	-	-	Sep 2009	192
Concord, MA	42.46	Aug 1997	55	-	-
Middlefield, CT*	41.52	Aug 1997	55	Sep 2009	132
Riverhead, NY	40.92	-	-	Aug 2009	54
Princeton, NJ	40.35	-	-	Jun 2009	66
Media, PA	39.92	-	-	Jun-Nov 2009-11	113.7 average
Churchville, MD*	39.56	Aug 1997	55	Oct 2010	280
Charlottesville, VA	38.03	-	-	Sep 2010	206
Richmond, VA	37.53	Aug 1997	55	-	-
Smithfield, NC*	35.51	Aug 1997	55	Jul 2010	63
Eutawville, SC*	33.39	Aug 1997	55	Jun 2010	74
Hahira, GA	30.99	-	-	Nov 2010	54
Jacksonville, FL*	30.32	Nov 1997	55	Oct 2010	110
Merritt Island, FL	28.36	Dec 1997	55	-	-
Venus, FL	27.07	-	-	Aug 2010	42
Homestead, FL*	25.47	Dec 1997	55	Jul 2010	84

[1] Details of the definition of # chromosomes are given in Cogni et al 2013 and Sezgin et al 2004).

* Indicates sites samples in both years at the same locality.

Supplemental Figure 1

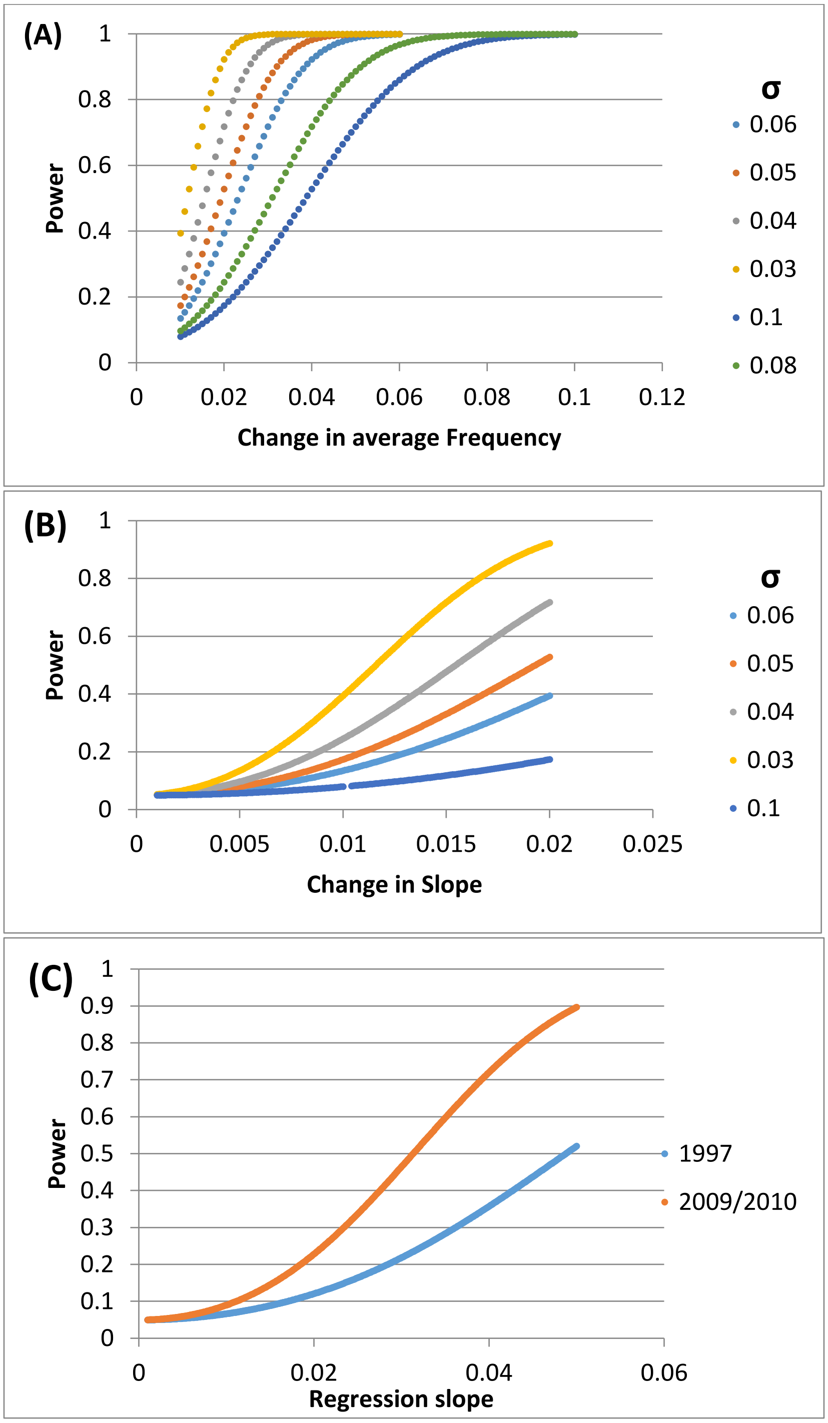

Figure S1. Results of power analyses on the linear regressions used to test changes in overall SNP frequency between years (A), changes in slope between years (B), and significant clines for individual SNPs within a year (C). In (A) and (B) different color represent simulations for different standard deviation values. In (C) a fixed value of standard deviation was used (the average 0.053531). The difference in power between years in (C) reflects differences in the number of populations (10 in 1997 *vs*. 18 in 2009/2010). The fact that we found many significant clines in the 1997 samples, even with reduced power, indicates that clines in metabolic genes are pervasive.

Supplemental Figure 2

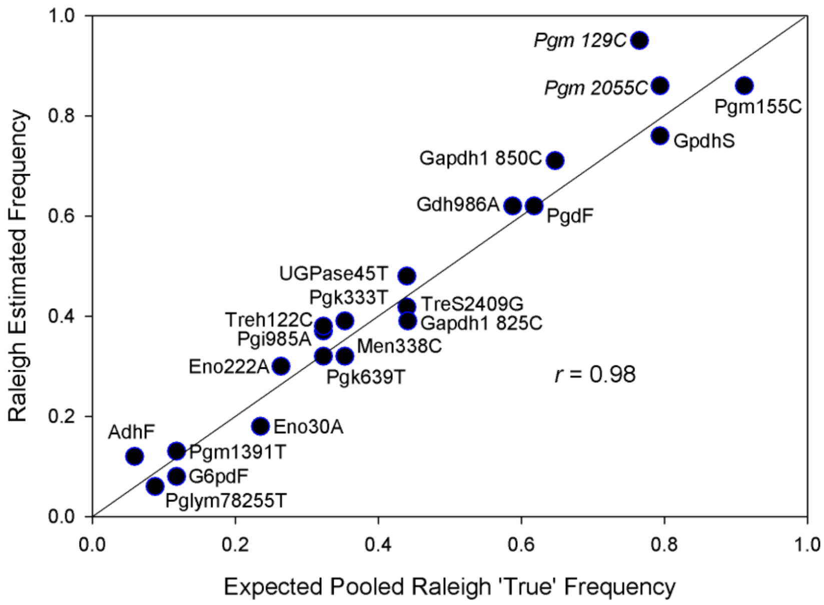

Figure S2. Plot of SNP frequencies estimated via bulk pyrosequencing for the DGRP (Raleigh) sample and the true frequency of the SNP expected from the Raleigh sequence data.
